# MiR-27b-3p inhibits the progression of renal fibrosis via suppressing STAT1

**DOI:** 10.1007/s13577-020-00474-z

**Published:** 2021-01-17

**Authors:** Lin Bai, Yongtao Lin, Juan Xie, Yiyuan Zhang, Hongwu Wang, Donghui Zheng

**Affiliations:** 1 Department of Nephrology, Affiliated Huai’an Hospital of Xuzhou Medical University, 62# Huaihai South Road, Huai’an, 223001 Jiangsu People’s Republic of China; 2 Xuzhou Medical University, Xuzhou, 221004 Jiangsu People’s Republic of China

**Keywords:** Chronic kidney disease, Renal fibrosis, miR-27b-3p, Unilateral ureteral obstruction

## Abstract

Renal fibrosis is a pathologic change in chronic kidney disease (CKD). MicroRNAs (miRNAs) have been shown to play an important role in the development of renal fibrosis. However, the biological role of miR-27b-3p in renal fibrosis remains unclear. Thus, this study aimed to investigate the role of miR-27b-3p in the progression of renal fibrosis. In this study, HK-2 cells were stimulated with transforming growth factor (TGF)-β1 for mimicking fibrosis progression in vitro. The unilateral ureteric obstruction (UUO)-induced mice renal fibrosis in vivo was established as well. The results indicated that the overexpression of miR-27b-3p significantly inhibited epithelial-to-mesenchymal transition (EMT) in TGF-β1-stimulated HK-2 cells, as shown by the decreased expressions of α-SMA, collagen III, Fibronectin and Vimentin. In addition, overexpression of miR-27b-3p markedly decreased TGF-β1-induced apoptosis in HK-2 cells, as evidenced by the decreased levels of Fas, active caspase 8 and active caspase 3. Meanwhile, dual-luciferase assay showed that miR-27b-3p downregulated signal transducers and activators of transcription 1 (STAT1) expression through direct binding with the 3′-UTR of STAT1. Furthermore, overexpression of miR-27b-3p attenuated UUO-induced renal fibrosis via downregulation of STAT1, α-SMA and collagen III. In conclusion, miR-27b-3p overexpression could alleviate renal fibrosis via suppressing STAT1 in vivo and in vitro. Therefore, miR-27b-3p might be a promising therapeutic target for the treatment of renal fibrosis.

## Introduction

Chronic kidney disease (CKD) is a common renal disease, which is characterized by a gradual loss of kidney function over a period of months or years [1]. Clinically, several complications are associated with CKD, including nutrition, anemia, osteodystrophy, hypertension and cardiovascular disease [2]. Evidence has shown that renal fibrosis is at the core of the high morbidity and mortality rates related to complications of various renal diseases [3]. In addition, renal fibrosis is the most common consequence of CKD, which could cause renal failure and renal collapse [4]. Meanwhile, epithelial-to-mesenchymal transition (EMT) is considered to be a crucial process of renal fibrosis [5]. During the process of EMT, renal epithelial cells acquire the mesenchymal phenotypes including α-SMA, and loss the epithelial phenotypes including E-cadherin [6]. However, there are currently no effective biomarkers available for assessment of CKD. Therefore, identification of promising renal fibrosis-related markers may help to understand the pathogenesis of CKD.

MicroRNAs (miRNAs) are a type of short, noncoding RNAs comprising 19–22 nucleotides [7]. It has been shown that miRNAs could regulate protein-coding gene expression through translational repression or mRNA degradation [8]. In addition, miRNAs could regulate the processes of cellular homeostasis, such as survival, proliferation, apoptosis and metabolism [9]. Moreover, miRNAs have been shown to be important players during the progression of renal fibrosis [10–15]. Jiang et al. indicated that miR-342 could alleviate renal fibrosis in diabetic nephropathy [16]. Morizane et al. found that miR-34c could inhibit renal fibrosis via suppressing the EMT process in mice with UUO [17]. Meanwhile, miR-27 was founded to be an anti-fibrotic miRNA, which could inhibit pulmonary fibroblast activation by targeting TGFβ receptor 1 and SMAD2 [18]. Lv et al. indicated that the overexpression of miR-27 could alleviate atrial fibrosis by the regulation of wnt/β-catenin signaling pathway [19]. Conserva et al. reported that the expression of miR-27b-3p was significantly downregulated in kidney tissues in patients with diabetic nephropathy [20]. However, the biological role of miR-27b-3p in renal fibrosis remains unclear. Therefore, this study aimed to investigate whether miR-27b-3p is a promising target for the development of therapeutics for renal fibrosis.

## Materials and methods

### Cell culture and cell transfection

Immortalized proximal tubule epithelial cell line (HK-2) was obtained from American Type Culture Collection (ATCC, Rockville, MD, USA). HK-2 cells were maintained in complete media (DMEM medium supplemented with 10% fetal bovine serum, 100 U/ml penicillin, 100 μg/ml streptomycin) and incubated in 5% CO_2_ at 37 °C.

MiR-27b-3p agonist and negative control (NC) were obtained from RiboBio (Guangzhou, China). HK-2 cells were transfected with 10 nM miR-27b-3p agonist, or NC using Lipofectamine 2000 reagent (Thermo Fisher Scientific, Waltham, MA, USA) for 24 h according to the manufacturer’s instructions.

### Lentivirus production and stable cell line construction

The STAT1 sequence was synthesized by GenePharma (Shanghai, China), and then inserted into the pLVX-IRES-PURO lentiviral expression vector plasmids (GenePharma). Then, 293T cells were transfected with lenti-STAT1 (STAT1-OE) vector plasmids and packaging plasmids (pLP/VSVG, pLP1, pLP2) for 72 h. After that, virus-containing supernatants were collected and added into HK-2 cells in the presence of polybrene (6 μg/ml). After 72 h of transduction, stable STAT1 overexpressed cells were then selected with 2.5 μg/ml puromycin.

### Reverse transcription-quantitative polymerase chain reaction (RT-qPCR)

HK-2 cells were transfected with miR-27b-3p agonist for 6 h, and then exposed to 5 ng/ml TGF-β1 for 48 h. Total RNA samples were extracted from HK-2 cells using the TRIpure Total RNA Extraction Reagent (ELK Biotechnology, Wuhan, China) according to the manufacturer's instructions. For reverse transcription, RNAs were reversely transcribed into cDNAs using the EntiLink™ 1st Strand cDNA Synthesis Kit (ELK Biotechnology). After that, qPCR analysis was performed using the SYBR^®^ Premix Ex Taq™ II (Takara Bio Inc. Shiga, Japan). The qPCR conditions were as follows: 95 °C for 3 min and 40 cycles of 95 °C for 10 s, 58 °C for 30 s, and 72 °C for 30 s. U6 and GAPDH were used as the internal control for normalizing miR-27b-3p and STAT1 expressions, respectively. The primer sequences were U6: forward, 5′-CTCGCTTCGGCAGCACAT-3′; reverse: 5′-AACGCTTCACGAATTTGCGT-3′. MiR-27b-3p: forward, 5′-AGTGGCTAAGTTCTGCCTCAAC-3′; reverse: 5′-CTCAACTGGTGTCGTGGAGTC-3′. GAPDH: forward, 5′-CATCATCCCTGCCTCTACTGG-3′; reverse: 5′-GTGGGTGTCGCTGTTGAAGTC-3′. STAT1: forward, 5′-ACTTTCCCTGACATCATTCGC-3′; reverse: 5′-TCTACAGAGCCCACTATCCGAG-3′.

### Western blot

Total proteins were quantified using BCA method (Beyotime Institute of Biotechnology, Shanghai, China). After that, proteins (30 μg proteins per lane) were separated on 10% sodium dodecyl sulfate-polyacrylamide gel electrophoresis, and then transferred onto polyvinylidene difluoride (PVDF) membrane (Thermo Fisher Scientific). After blocking with 5% skimmed milk in TBST for 1 h, the membrane was incubated with primary antibodies against α-SMA (1:1000, Abcam), Collagen III (1:1000, Abcam), Fibronectin (1:1000, Abcam), Vimentin (1:1000, Abcam), p-STAT1 (1:1000, Abcam), STAT1 (1:1000, Abcam), Fas (1:1000, Abcam), Active caspase 8 (1:1000, Abcam), Active caspase 3 (1:1000, Abcam), and GAPDH (1:1000, Abcam) at 4 °C overnight. Subsequently, the membrane was incubated with secondary antibodies (1:5000, Abcam) for 1 h at room temperature, and bands were detected with the ECL Chemiluminescent Substrate Reagent Kit (Thermo Fisher Scientific). GAPDH was acted as the internal control.

### Dual-luciferase reporter assay

The cDNA fragments containing the predicted miR-27b-3p binding sites were sub-cloned into pmirGLO dual-luciferase vector, named wild-type (WT)-STAT1 or mutant (MT)-STAT1. After that, HK-2 cells were co-transfected with plasmids (WT-STAT1 or MT-STAT1) and miR-27b-3p agonist, respectively using Lipofectamine 2000. Later on, the luciferase activity in cell lysate was detected using the Dual-Luciferase Reporter Assay System (Promega, Madison, WI, USA) at 48 h according to the manufacturer’s protocol.

### Flow cytometry

HK-2 cells were washed three times with PBS, and then stained with 5 μL of propidium iodide (PI, Sigma-Aldrich, St. Louis, MO, USA) and 5 μL of Annexin V-FITC (Sigma-Aldrich) at room temperature for 15 min. Subsequently, the percentage of apoptotic cells was assessed using the flow cytometer (FACScan™, BD Biosciences, Franklin Lakes, NJ, USA) with BD FACSDiva software.

### Animal study

C57BL/6 mice (8 weeks old, 20–25 g) were purchased from the Chinese Academy of Sciences Experiment Center (Shanghai, China). All animal experiments were approved by the Institutional Ethical Committee of the Affiliated Huai'an Hospital of Xuzhou Medical University. We confirmed that ethical and legal approval was obtained prior to the commencement of the study. Meanwhile, all experiments were performed according to the National Institutes of Health Guide for the Care and Use of laboratory animals. The animals were randomly divided into four groups (*n* = 5): blank, unilateral ureteral occlusion (UUO), UUO + NC, UUO + miR-27b-3p agonist groups. The UUO model was established using an established procedure [21]. Briefly, mice were anesthetized with 50 mg/kg pentobarbital (i.p.). After an abdominal midline laparotomy, the left ureter was exposed. After that, the left ureter was obstructed using 2-point ligations with silk sutures (4-0), and the incision was closed in layers. Sham animals underwent the abdominal midline laparotomy, but the left ureter was not obstructed. MiR-27b-3p agonist (50 nM, RiboBio, Guangzhou, China) was administered via tail vein injection every day as previously described [22]. Later on, animals were sacrificed in 4 weeks, and the renal tissues were collected.

### Histology analysis

Kidney tissues were fixed in 4% paraformaldehyde, and then embedded in paraffin. The specimens (5 μm) were stained with haematoxylin–eosin (H&E), periodic acid–Schiff reagent (PAS) or Masson’s trichrome staining to determine the degree of renal fibrosis. The slides were observed using a laser scanning confocal microscope (LSM, Carl Zeiss). Quantification of the fibrotic area was carried out using Image-Pro software (Bethesda, MD, USA). Integrity of the glomerulotubular junction and proximal tubular mass was examined in *Lotus tetragonolobus* lectin (LTL)-stained kidney sections as described previously [23].

### ELISA

The urea and creatinine assay kits (Nanjing Jiancheng Bioengineering Institute, Jiangsu, China) were used to detect the levels of blood urea nitrogen (BUN) and creatinine (CR) in urine of mice, respectively, according to the manufacturer’s protocol.

### Statistical analysis

All data were repeated in triplicate. Data are presented as the mean ± SD. All statistical analyses were performed using GraphPad Prism software (version 7.0, La Jolla, CA, USA). One-way analysis of variance (ANOVA) and Tukey’s tests were carried out for multiple group comparisons. For the comparison of two groups, Student’s *t* test was applied. Differences were considered to be significant at *p < 0.05.

## Results

### Overexpression of miR-27b-3p inhibited TGF-β1-induced fibrosis in HK-2 cells

**Fig. 1:**
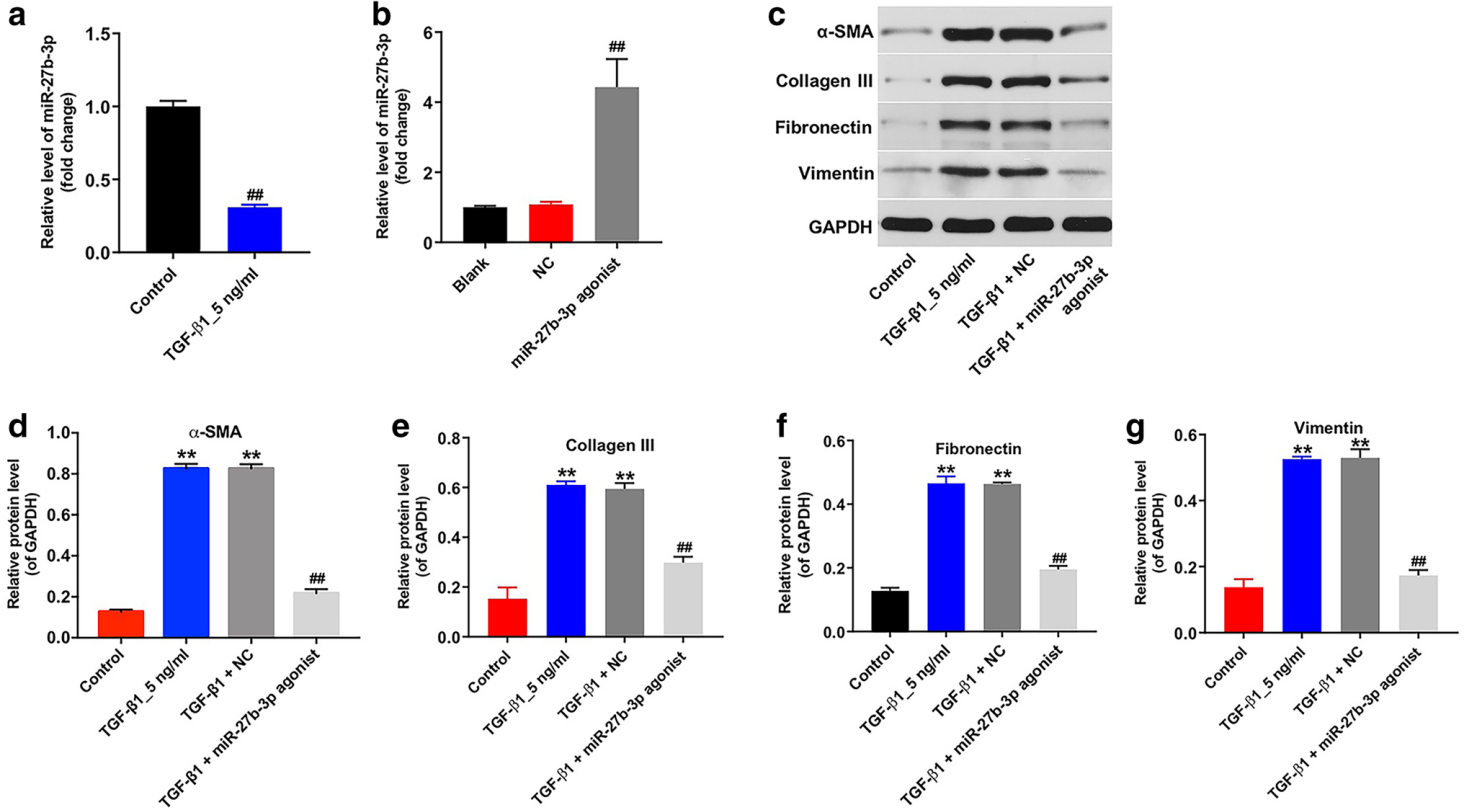
Overexpression of miR-27b-3p attenuated TGF-β1-mediated EMT in HK-2 cells. **a** HK-2 cells were treated with 5 ng/ml TGF-β1 for 48 h. RT-qPCR was used to measure the level of miR-27b-3p in HK-2 cells. **b** The level of miR-27b-3p in HK-2 cells transfected with miR-27b-3p agonist was detected using qRT-PCR. **c** HK-2 cells were transfected with miR-27b-3p agonist for 6 h, and then exposed to 5 ng/ml TGF-β1 for 48 h. Expressions of α-SMA, Collagen III, Fibronectin and Vimentin in HK-2 cells were detected with western blotting. Images are representative of three independent experiments. **d**–**g** The relative expressions of α-SMA, Collagen III, Fibronectin and Vimentin in HK-2 cells were quantified via normalization to GAPDH. **p < 0.01, compared with the control group. ^##^*p* < 0.01, compared with the TGF-β1 group

Evidences have shown that TGF-β1 is the most important inducer of EMT, which plays an important role in the development of tissue fibrosis [24, 25]. In this study, HK-2 cells were treated with TGF-β1 (5 ng/ml) for 48 h and the expression of miR-27b-3p in HK-2 cells was detected by RT-qPCR. As shown in Fig. 1a, TGF-β1 significantly decreased the level of miR-27b-3p in HK-2 cells. In addition, the level of miR-27b-3p was markedly upregulated in HK-2 cells following transfection with miR-27b-3p agonist (Fig. 1b). Moreover, TGF-β1 stimulation notably upregulated the levels of EMT-related proteins α-SMA, Collagen III, Fibronectin and Vimentin in HK-2 cells; however, these levels were remarkably decreased by miR-27b-3p overexpression (Fig. 1c–g). These data indicated that overexpression of miR-27b-3p could inhibit TGF-β1-induced fibrosis in HK-2 cells via suppressing the EMT process.

### STAT1 was a binding target of miR-27b-3p

**Fig. 2:**
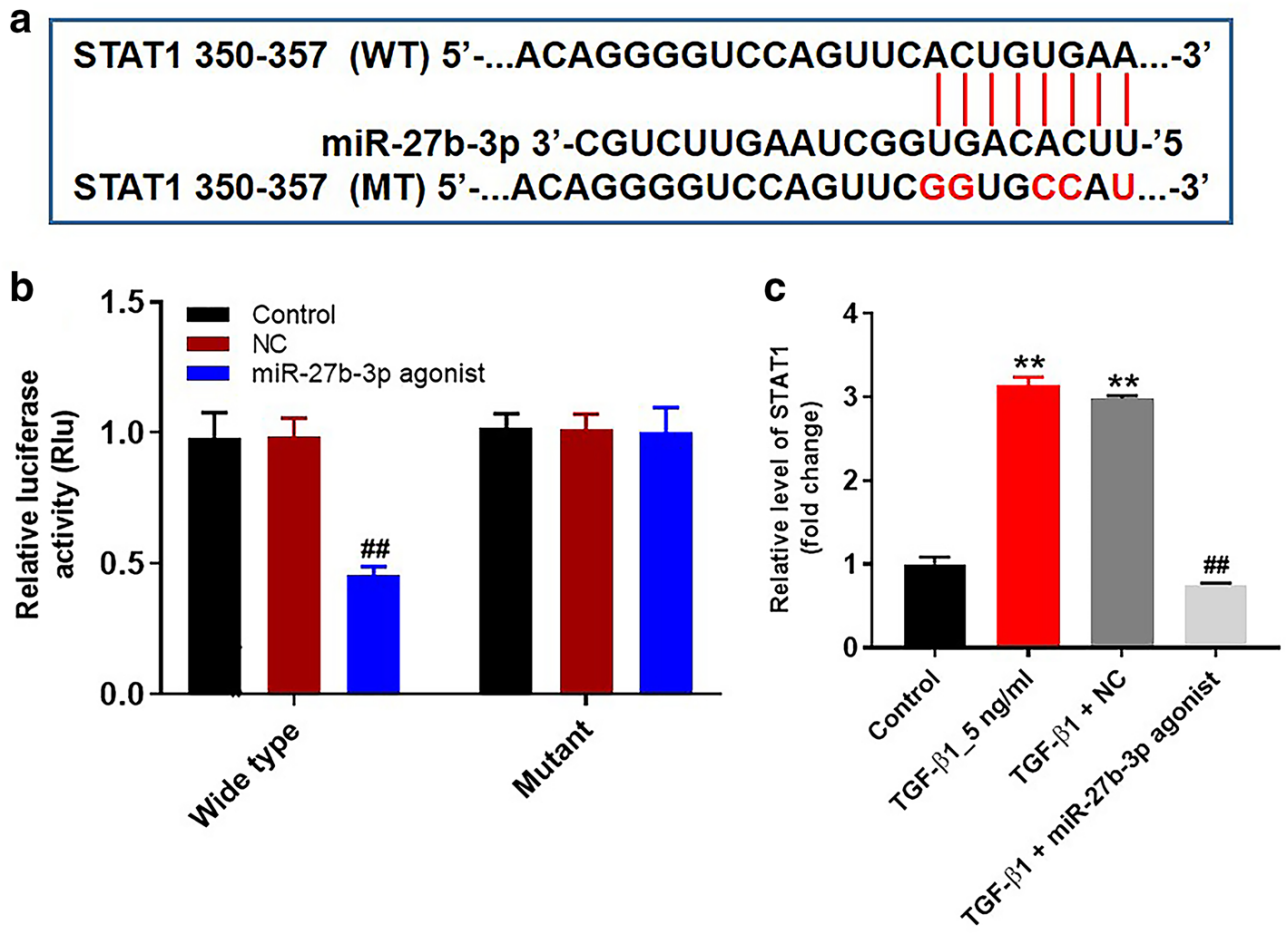
STAT1 was a binding target of miR-27b-3p. **a** Sequence alignment of miR-27b-3p with the binding sites within the WT or MT regions of STAT1. **b** The luciferase activity in HK-2 cells following co-transfecting with STAT1-WT/MT 3′-UTR plasmid and miR-27b-3p agonist were detected using dual-luciferase reporter assay. **c** HK-2 cells were transfected with miR-27b-3p agonist for 6 h, and then exposed to 5 ng/ml TGF-β1 for 48 h. The level of STAT1 in HK-2 cells was detected by RT-qPCR. **p < 0.01, compared with the control group. ^##^*p* < 0.01, compared with the TGF-β1 group

It has been shown that miRNAs could regulate gene expression through directly binding to 3′ untranslated region (UTR) of mRNAs [8]. TargetScan dataset (http://www.targetscan.org/vert_71/) predicted that STAT1 might be a potential target of miR-27b-3p (Fig. 2a). In addition, dual-luciferase reporter assay validated that miR-27b-3p agonist significantly decreased the luciferase activity in HK-2 cells co-transfected with STAT1-WT, while miR-27b-3p agonist had no effect on luciferase activity in HK-2 cells co-transfected with STAT1-MT (Fig. 2b). Meanwhile, overexpression of miR-27b-3p notably decreased protein level of STAT1 in TGF-β1-treated HK-2 cells (Fig. 2c). Collectively, STAT1 was a binding target of miR-27b-3p.

### Overexpression of miR-27b-3p inhibited TGF-β1-induced apoptosis in HK-2 cells

**Fig. 3:**
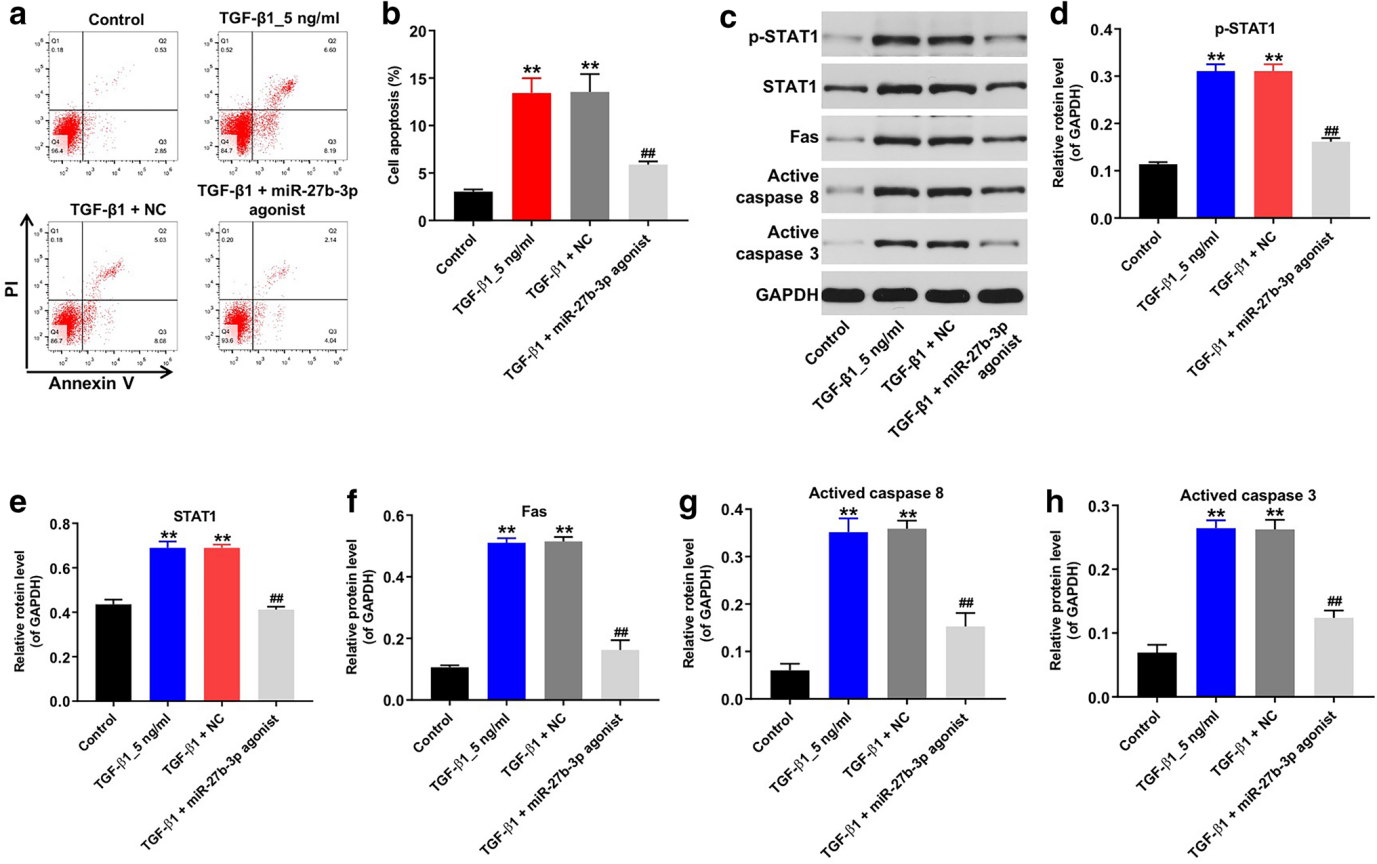
Overexpression of miR-27b-3p inhibited TGF-β1-induced apoptosis in HK-2 cells. HK-2 cells were transfected with miR-27b-3p agonist for 6 h, and then exposed to 5 ng/ml TGF-β1 for 48 h. **a**, **b** Apoptotic cells were detected with Annexin V and PI double staining. **(C)** Expressions of p-STAT1, STAT1, Fas, active caspase 8, active caspase 3 in HK-2 cells were detected with western blotting. Images are representative of three independent experiments. **d**–**h** The relative expressions of p-STAT1, STAT1, Fas, active caspase 8, active caspase 3 in HK-2 cells were quantified via normalization to GAPDH. **p < 0.01, compared with the Control group. ^##^*p* < 0.01, compared with the TGF-β1 group

It has been shown that renal fibrosis is related to enhanced tubular cell apoptosis [26]. Then flow cytometry assay was performed to detect cell apoptosis. As shown in Fig. 3a, b, TGF-β1 notably induced apoptosis of HK cells, while TGF-β1-induced apoptosis was significantly reversed by miR-27b-3p overexpression. In addition, the expressions of p-STAT1, STAT1, Fas, active caspase 8 and active caspase 3 in HK-2 cells were detected by western blot assay. As indicated in Fig. 3c–h, TGF-β1 markedly upregulated the expressions of p-STAT1, STAT1, Fas, active caspase 8 and active caspase 3 in HK-2 cells; however, these TGF-β1-induced changes were obviously reversed by miR-27b-3p agonist. These data suggested that overexpression of miR-27b-3p could inhibit TGF-β1-induced apoptosis in HK-2 cells.

### Overexpression of miR-27b-3p alleviated renal fibrosis in UUO mice

**Fig. 4:**
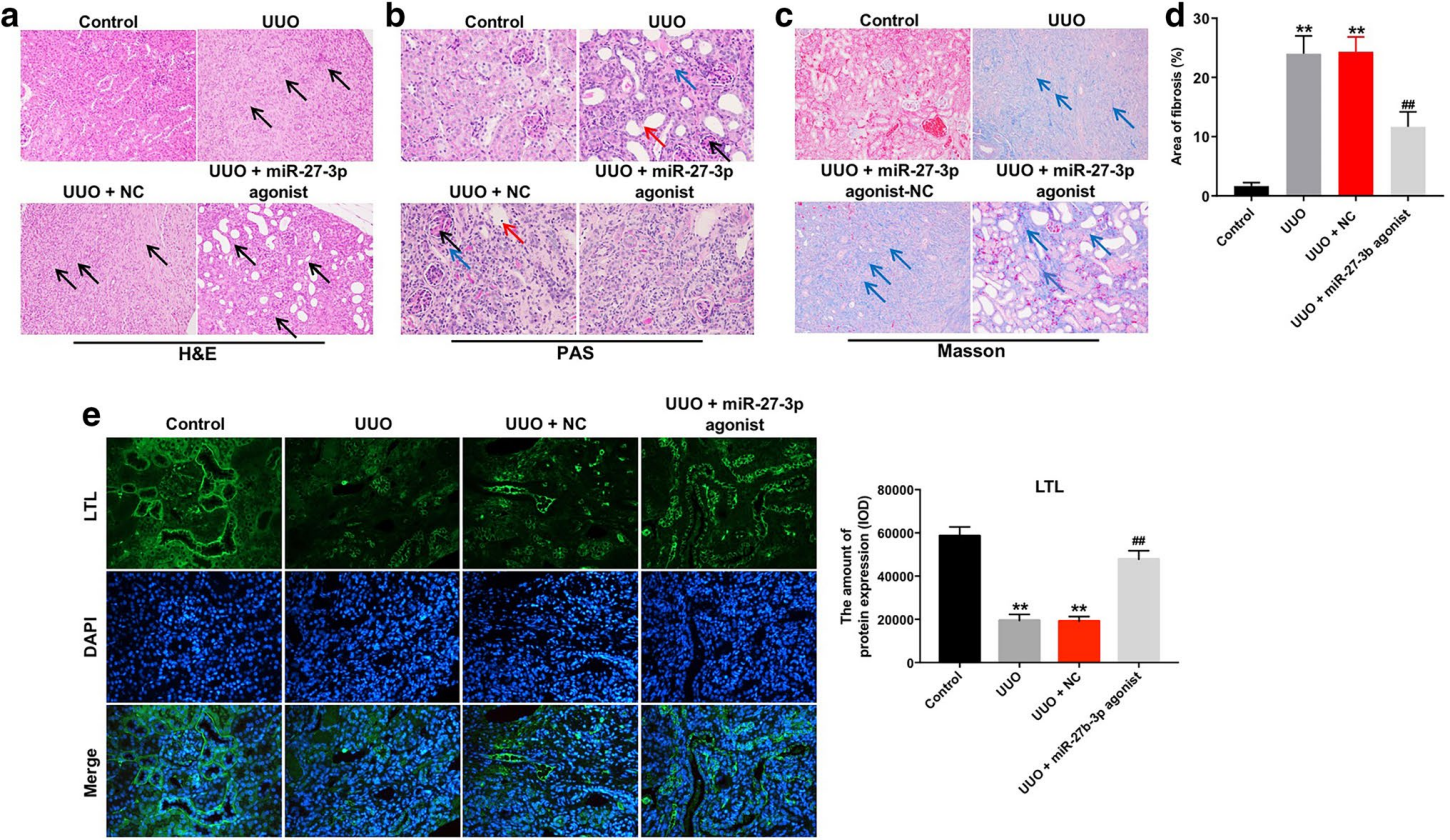
Overexpression of miR-27b-3p alleviated renal fibrosis in vivo. **a** Analysis of kidney injury in UUO kidneys by H&E staining (magnification, × 200). black arrows pointed to fibroblasts. **b** Representative photomicrographs of PAS-stained kidney sections (magnification, × 400). Black arrows pointed to glomerular mesangial matrix; red arrows pointed to exfoliation of renal tubular epithelial cells; blue arrows pointed to renal interstitial edema. **c** Analysis of collagen deposition and renal fibrosis by Masson’s trichrome staining (magnification, × 200). Blue arrow pointed to collagen fibers. **d** Total lung fibrotic area was measured by Image-Pro Plus. **e** Integrity of the glomerulotubular junction and proximal tubular mass were determined in LTL staining (magnification, × 200). **p < 0.01, compared with the control group. ^##^*p* < 0.01, compared with the UUO group

Next, to investigate the role of miR-27b-3p in renal fibrosis in vivo, UUO mouse model was established. H&E staining assay indicated that interstitial expansion was observed in obstructed kidneys after UUO, while overexpression of miR-27b-3p reduced the expansion of interstitial area in UUO mice (Fig. 4a). In addition, PAS staining assay showed that renal interstitial edema, exfoliation of renal tubular epithelial cells and increased glomerular mesangial matrix were observed in obstructed kidneys after UUO; however, these changes were markedly alleviated by miR-27b-3p agonist treatment (Fig. 4b). Moreover, Masson’s trichrome staining assay indicated that overexpression of miR-27b-3p obviously reduced the fibrotic area in obstructed kidneys after UUO (Fig. 4c, d). Meanwhile. LTL staining revealed that the fraction of glomerulotubular junctions and proximal tubule were reduced in the obstructed kidney of in UUO mice; however, these changes were reversed by miR-27b-3p agomir (Fig. 4e). These data indicated that overexpression of miR-27b-3p could alleviate renal fibrosis in vivo.

**Fig. 5:**
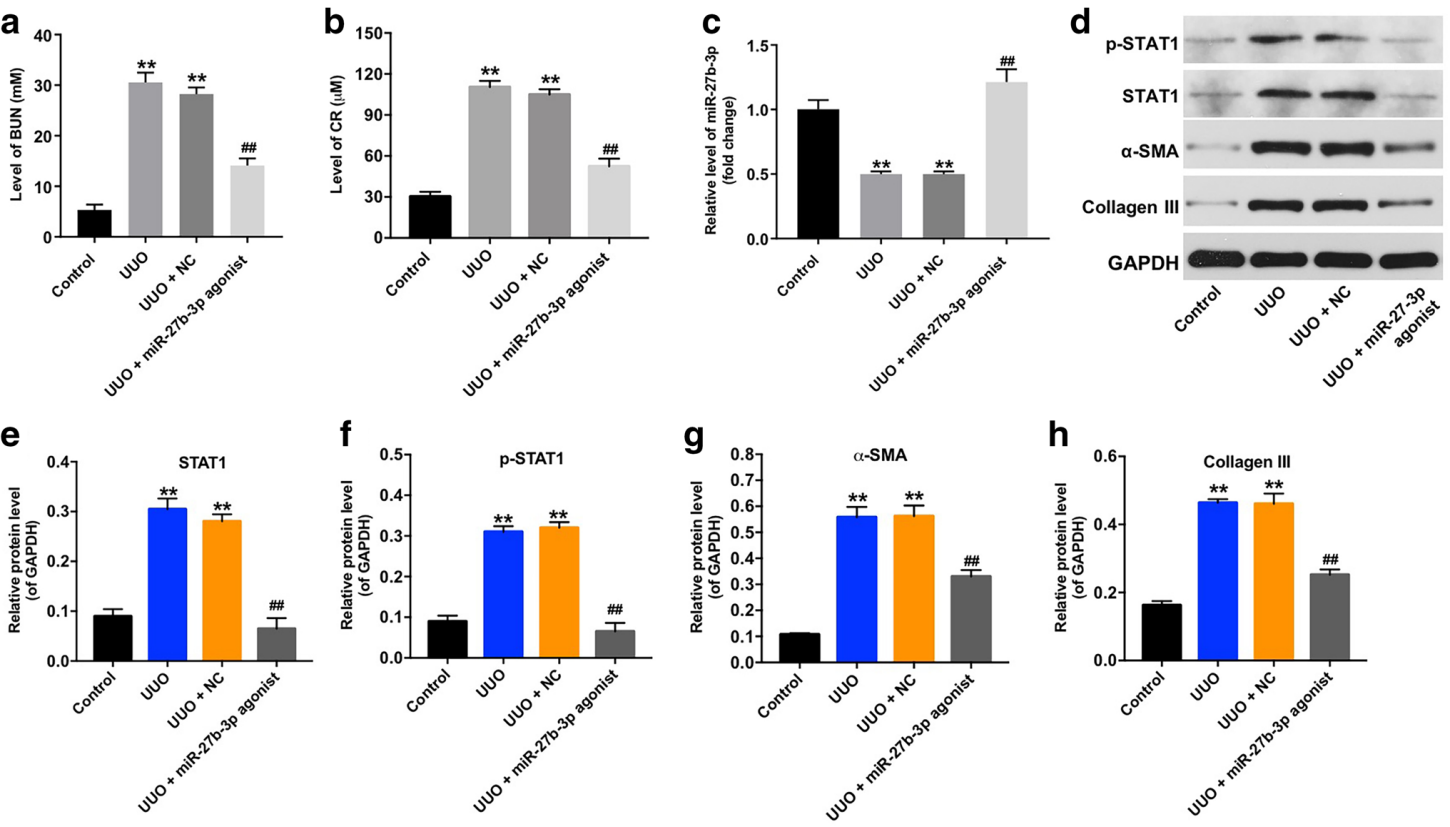
Overexpression of miR-27b-3p alleviated renal fibrosis in vivo. **a** The level of BUN in urine of mice was detected by ELISA. **b** The level of CR in urine of mice was detected by ELISA. **(C)** RT-qPCR analysis showed the level of miR-27b-3p in kidney tissues in UUO mice model. **d** Expression levels of p-STAT1, STAT1, α-SMA, Collagen III in kidney tissues were detected with western blotting. Images are representative of three independent experiments. **e**–**h** The relative expressions of p-STAT1, STAT1, α-SMA, Collagen III in kidney tissues were quantified via normalization to GAPDH. **p < 0.01, compared with the control group. ^##^*p* < 0.01, compared with the UUO group

Furthermore, the levels of BUN and CR in urine of mice were significantly increased after UUO; consistently, these phenomena were significantly reversed by miR-27b-3p agonist (Fig. 5a, b). Moreover, the level of miR-27b-3p was notably decreased in obstructed kidneys in UUO mice; however, overexpression of miR-27b-3p markedly upregulated the level of miR-27b-3p in kidneys (Fig. 5c). Meanwhile, the expressions of p-STAT1, STAT1, α-SMA, and Collagen III were significantly upregulated in obstructed kidneys in UUO mice, while overexpression of miR-27b-3p reversed these changes (Fig. 5d–h). All these results illustrated that overexpression of miR-27b-3p could alleviate renal fibrosis in vivo via suppressing STAT1.

### Overexpression of miR-27b-3p inhibited TGF-β1-induced apoptosis in HK-2 cells via suppressing STAT1

**Fig. 6:**
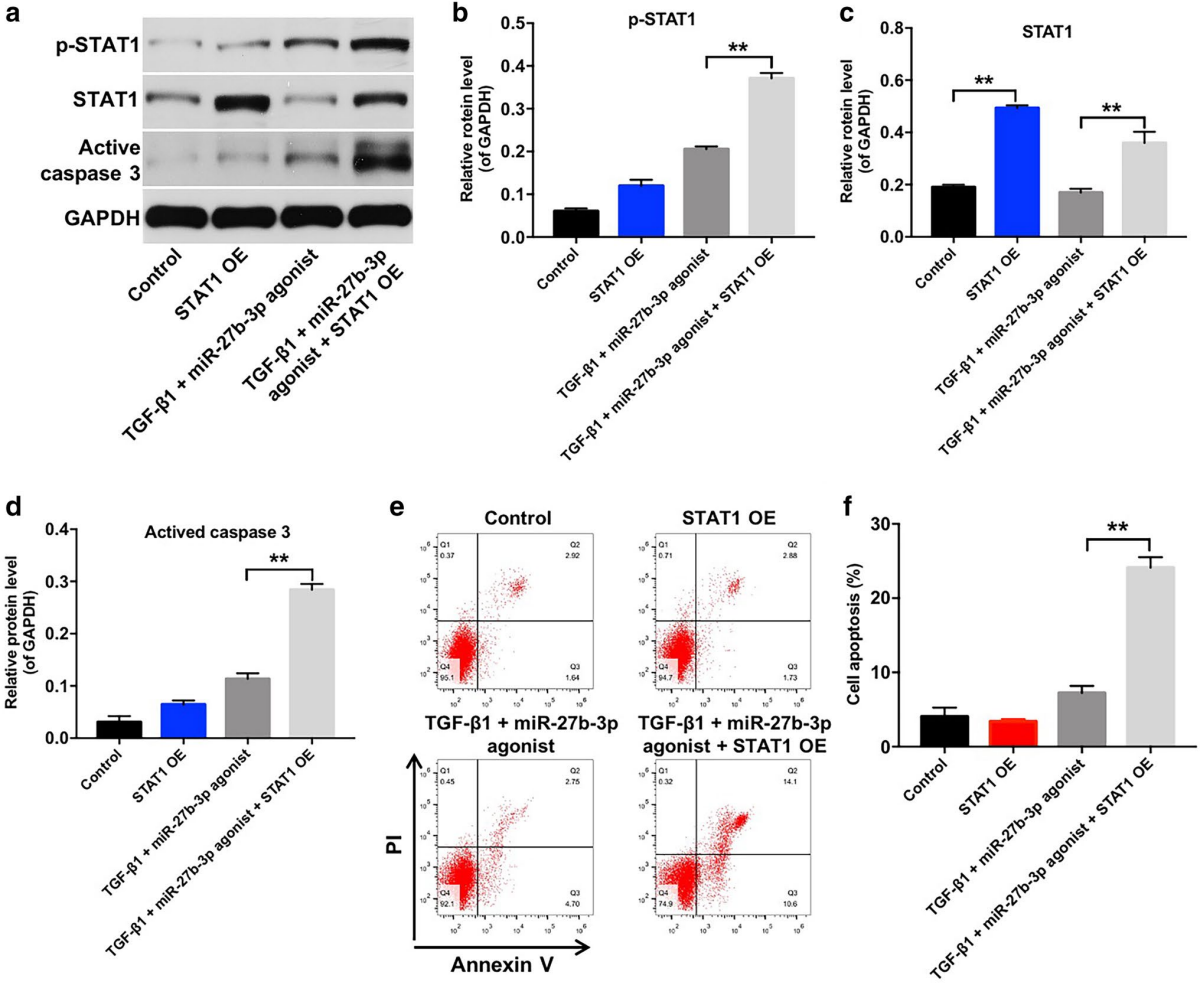
Overexpression of miR-27b-3p inhibited TGF-β1-induced apoptosis in HK-2 cells via inhibiting STAT1. **a**–**d** Western blot analysis of p-STAT1, STAT1 and active caspase 3 expressions in HK-2 cells treated with 5 ng/ml TGF-β1, STAT1 OE and miR-27b-3p agonist. **e**, **f** Apoptotic cells were detected with Annexin V and PI double staining. **p < 0.01

Finally, we observed that overexpression of STAT1 resulted in increased expression of STAT1 in TGF-β1-treated HK-2 cells, while overexpression of STAT1 did not affect the levels of p-STAT1 and active caspase 3 in HK-2 cells (Fig. 6a–d). Interestingly, HK-2 cells incubated with TGF-β1 and miR-27b-3p agonist showed significantly reduced p-STAT1, STAT1 and active caspase 3 protein levels compared with HK-2 cells incubated with 5 ng/ml TGF-β1; however, STAT1 overexpression markedly increased p-STAT1, STAT1 and active caspase 3 protein levels in HK-2 cells incubated with TGF-β1 and miR-27b-3p agonist (Figs. 3a–c, f, 6–d). Meanwhile, overexpression of STAT1 caused no change in apoptosis, while the inhibitory effect of miR-27b-3p agonist on apoptosis in TGF-β1-treated HK-2 cells was reversed by STAT1 overexpression (Figs. 3g, h, 6e, f). These results indicated that overexpression of miR-27b-3p could inhibit TGF-β1-induced apoptosis in HK-2 cells via suppressing STAT1.

## Discussion

Renal fibrosis is recognized as the most common pathological characteristic of CKD, which could result in end-stage renal disease [27]. However, the molecular pathogenesis of renal fibrosis remains not fully discovered. Recently, it has been shown that several miRNAs participated in the development of renal fibrosis [28]. In this study, we found that the level of miR-27b-3p was significantly decreased in TGF-β1-treated HK-2 cells and in obstructed kidneys of UUO mice. In addition, miR-27b-3p overexpression could attenuate renal fibrosis in vitro and in vivo. These data suggested that miR-27b-3p might function as a key regulator during the development of renal fibrosis.

EMT plays an important role in the progress of renal fibrosis, which is associated with the formation of fibroblasts and myofibroblasts in kidney disease [29]. It has been shown that suppressing EMT could attenuate renal fibrosis [30]. In addition, TGF-β1 has been functioned as a vital regulator of EMT and tissue fibrosis [31]. TGF-β1 could induce the expressions of mesenchymal markers, including α-SMA, Collagen III, and Fibronectin [32, 33]. Wang et al. found that let-7d significantly attenuated TGF-β1-induced fibrogenesis in kidney tubular epithelial cells via inhibiting EMT [34]. Wu et al. reported that miR-145 contribute to fibrosis via promoting EMT process [35]. In this study, we found that overexpression of miR-27b-3p significantly inhibited TGF-β1-induced EMT in HK-2 cells via downregulation of α-SMA, Collagen III, Fibronectin and Vimentin. However, increasing studies demonstrated EMT in vivo was controversial, they viewed that renal epithelial cells do not directly contribute to interstitial myofibroblast cells in vivo [36, 37]. In contrast, oba et al. indicated that miR-200 could ameliorate tubulointerstitial fibrosis in the kidneys of UUO mice via inhibiting EMT [38]. Meanwhile, we found that overexpression of miR-27b-3p inhibited renal fibrosis in UUO mice via downregulation of α-SMA and Collagen III. These data indicated that overexpression of miR-27b-3p could attenuate renal fibrosis via inhibiting EMT in vitro and in vivo.

Evidence has demonstrated that miRNAs exert biological functions through suppressing the expression of their target genes [39]. Previous study indicated that miR-27b-3p could inhibit the metastasis of endometrial cancer cells via inhibiting MARCH7 and suppressing EMT [40]. To investigate the underlying mechanism of miR-27b-3p in the progression of renal fibrosis, bioinformatics analysis tool TargetScan and luciferase reporter assay were applied to identify potential target gene of miR-27b-3p. These data indicated that STAT1 was a potential binding target of miR-27b-3p. Evidences have shown that STAT1 played important roles in inflammatory response and tumorigenesis [41, 42]. Zhang et al. found that inhibition of STAT1 could suppress the progress of liver fibrosis [43]. In addition, STAT signaling plays a vital role in renal fibrosis, activation of STAT could induce EMT and contribute to renal damage [44, 45]. In this study, we observed that TGF-β1 markedly increased the expressions of p-STAT1 and STAT1 in HK-2 cells, which was consistent with previous study [46]. Significantly, overexpression of miR-27b-3p decreased the expressions of p-STAT1 and STAT1 in TGF-β1-stimulated HK-2 cells. Moreover, overexpression of miR-27b-3p notably prevented the increased expressions of p-STAT1 and STAT1 in UUO kidney. These data indicated that overexpression of miR-27b-3p could attenuate renal fibrosis in vitro and in vivo via inhibiting STAT1. Zhang et al. indicated that miRNA-22 could promote renal fibrosis by targeting PTEN [47]. Zhu et al. indicated that miR-98-5p could alleviate renal fibrosis via targeting high mobility group A [48]. In this study, we found that miR-27b-3p could alleviate renal fibrosis via targeting STAT1. These results demonstrated a novel miR-27b-3p/STAT1 pathway in renal fibrosis.

In this study, TGF-β1 obviously induced the apoptosis in HK-2 cells. Previous study indicated that oxidative stress, ischemia, and hypoxia could lead to renal tissue injury after UUO [49]. Meanwhile, renal fibrosis is related to an increased tubular cell apoptosis [50]. Zhou et al. indicated that inhibition of apoptosis in renal tubular epithelial cells might be a promising approach for anti-fibrosis treatment [51]. In this study, we found that overexpression of miR-27b-3p could suppress TGF-β1-induced apoptosis in HK-2 cells. In addition, overexpression of miR-27b-3p could inhibit Fas-mediated apoptosis in TGF-β1-stimulated HK-2 cells via downregulation of Fas, active caspase 8, active caspase 3. Xu et al. found that activation of STAT1 could increase the expression of Fas [52]. These results illustrated that overexpression of miR-27b-3p could ameliorate renal fibrosis in TGF-β1-stimulated HK-2 cells via inactivating STAT1, and then suppressing Fas signaling. Dysregulation of apoptosis and EMT are linked with the process of renal fibrosis [53]. In this study, we found that overexpression of miR-27b-3p not only inhibited the apoptosis but also simultaneously suppressed the EMT in TGF-β1-stimulated HK-2 cells, indicating that both apoptosis and EMT are involved in miR-27b-3p-mediated regulation in renal fibrosis. Collectively, our findings provided evidence that overexpression of miR-27b-3p ameliorated renal fibrosis and restored renal function via inhibiting EMT and suppression of apoptosis in renal tubular epithelial cells.

Indeed, renal fibrosis is a progressive process that is the inevitable outcome of all progressive CKD. Although the treatment investigated here in mice produced a decline in renal fibrosis over a 4-week period, a question that remains to be answered is whether the beneficial effects of miRNA therapeutics are maintained in the long-term in a clinically mice model of UUO. Therefore, future research should focus on a longer term analysis. In addition, in this study, we only determined that miR-27b-3p could inhibit the apoptosis and EMT in TGF-β1-treated HK-2 cells by targeting STAT1. Thus, further study is needed to identify whether overexpression of miR-27b-3p inhibited the progression of renal fibrosis via targeting other genes.

## Conclusion

In this study, the results indicated that aberrant expression of miR-27b-3p plays an important role in the progression of renal fibrosis, and overexpression of miR-27b-3p could attenuate the progression of renal fibrosis through inhibition of STAT1 signaling. Therefore, miR-27b-3p might be a promising target for the treatment of renal fibrosis.

## References

[1] Li S, Lin Q, Shao X, Zhu X, Wu J, Wu B (2019). Drp1-regulated PARK2-dependent mitophagy protects against renal fibrosis in unilateral ureteral obstruction. Free Radical Biol Med.

[2] Thomas R, Kanso A, Sedor JR (2008). Chronic kidney disease and its complications. Prim Care.

[3] Wang W, Jia YJ, Yang YL, Xue M, Zheng ZJ, Wang L (2020). LncRNA GAS5 exacerbates renal tubular epithelial fibrosis by acting as a competing endogenous RNA of miR-96-5p. Biomed Pharmacother..

[4] Nogueira A, Pires MJ, Oliveira PA (2017). Pathophysiological mechanisms of renal fibrosis: a review of animal models and therapeutic strategies. In Vivo (Athens Greece).

[5] Zhou J, Jiang H (2019). Livin is involved in TGF-beta1-induced renal tubular epithelial-mesenchymal transition through lncRNA-ATB. Ann Transl Med.

[6] Hong W, Zhang G, Lu H, Guo Y, Zheng S, Zhu H (2019). Epithelial and interstitial Notch1 activity contributes to the myofibroblastic phenotype and fibrosis. Cell Commun Signal.

[7] Su Z, Jiang G, Chen J, Liu X, Zhao H, Fang Z (2020). MicroRNA-429 inhibits cancer cell proliferation and migration by targeting AKT1 in renal cell carcinoma. Mol Clin Oncol.

[8] Bouyssou JM, Manier S, Huynh D, Issa S, Roccaro AM, Ghobrial IM (2014). Regulation of microRNAs in cancer metastasis. Biochem Biophys Acta.

[9] Iqbal MA, Arora S, Prakasam G, Calin GA, Syed MA (2019). MicroRNA in lung cancer: role, mechanisms, pathways and therapeutic relevance. Mol Aspects Med.

[10] Loboda A, Sobczak M, Jozkowicz A, Dulak J (2016). TGF-β1/Smads and miR-21 in renal fibrosis and inflammation. Mediators inflamm.

[11] Lv W, Fan F, Wang Y, Gonzalez-Fernandez E, Wang C, Yang L (2018). Therapeutic potential of microRNAs for the treatment of renal fibrosis and CKD. Physiol Genom.

[12] Van der Hauwaert C, Glowacki F, Pottier N, Cauffiez C (2019). Non-coding RNAs as new therapeutic targets in the context of renal fibrosis. Int J Mol Sci..

[13] Ichii O, Horino T (2018). MicroRNAs associated with the development of kidney diseases in humans and animals. J Toxicol Pathol.

[14] Jaswani P, Prakash S, Dhar A, Sharma RK, Prasad N, Agrawal S (2017). MicroRNAs involvement in renal pathophysiology: a Bird's Eye view. Indian J Nephrol.

[15] Kota SK, Kota SB (2017). Noncoding RNA and epigenetic gene regulation in renal diseases. Drug Discov Today.

[16] Jiang ZH, Tang YZ, Song HN, Yang M, Li B, Ni CL (2019). miRNA342 suppresses renal interstitial fibrosis in diabetic nephropathy by targeting SOX6. Int J Mol Med.

[17] Morizane R, Fujii S, Monkawa T, Hiratsuka K, Yamaguchi S, Homma K (2014). miR-34c attenuates epithelial-mesenchymal transition and kidney fibrosis with ureteral obstruction. Sci Rep.

[18] Zeng X, Huang C, Senavirathna L, Wang P, Liu L (2017). miR-27b inhibits fibroblast activation via targeting TGFβ signaling pathway. BMC Cell Biol.

[19] Lv X, Li J, Hu Y, Wang S, Yang C, Li C (2019). Overexpression of miR-27b-3p Targeting Wnt3a Regulates the Signaling Pathway of Wnt/β-Catenin and Attenuates Atrial Fibrosis in Rats with Atrial Fibrillation. Oxid Med Cell Longev.

[20] Conserva F, Barozzino M, Pesce F, Divella C, Oranger A, Papale M (2019). Urinary miRNA-27b-3p and miRNA-1228-3p correlate with the progression of Kidney Fibrosis in Diabetic Nephropathy. Sci Rep.

[21] Li S, Wang Y, Chen L, Wang Z, Liu G, Zuo B (2019). Beraprost sodium mitigates renal interstitial fibrosis through repairing renal microvessels. J Mol Med.

[22] Krützfeldt J, Rajewsky N, Braich R, Rajeev KG, Tuschl T, Manoharan M (2005). Silencing of microRNAs in vivo with 'antagomirs'. Nature.

[23] Galarreta CI, Thornhill BA, Forbes MS, Simpkins LN, Kim DK, Chevalier RL (2013). Transforming growth factor-β1 receptor inhibition preserves glomerulotubular integrity during ureteral obstruction in adults but worsens injury in neonatal mice. Am J Physiol Renal Physiol.

[24] Kim MK, Maeng YI, Sung WJ, Oh HK, Park JB, Yoon GS (2013). The differential expression of TGF-beta1, ILK and wnt signaling inducing epithelial to mesenchymal transition in human renal fibrogenesis: an immunohistochemical study. Int J Clin Exp Pathol.

[25] Zhan J, Liu M, Pan L, He L, Guo Y (2019). Oxidative stress and TGF-beta1/Smads signaling are involved in *Rosa roxburghii* fruit extract alleviating renal fibrosis. Evid Based Complement Altern Med eCAM.

[26] Xu W, Shao X, Tian L, Gu L, Zhang M, Wang Q (2014). Astragaloside IV ameliorates renal fibrosis via the inhibition of mitogen-activated protein kinases and antiapoptosis in vivo and in vitro. J Pharmacol Exp Ther.

[27] Eddy AA (2014). Overview of the cellular and molecular basis of kidney fibrosis. Kidney Int Suppl.

[28] Yu J, Yu C, Feng B, Zhan X, Luo N, Yu X (2019). Intrarenal microRNA signature related to the fibrosis process in chronic kidney disease: identification and functional validation of key miRNAs. BMC Nephrol.

[29] Prunotto M, Budd DC, Gabbiani G, Meier M, Formentini I, Hartmann G (2012). Epithelial-mesenchymal crosstalk alteration in kidney fibrosis. J Pathol.

[30] Lovisa S, Zeisberg M, Kalluri R (2016). Partial epithelial-to-mesenchymal transition and other new mechanisms of kidney fibrosis. Trends Endocrinol Metab.

[31] Ha H, Yu MR, Lee HB (2001). High glucose-induced PKC activation mediates TGF-beta 1 and fibronectin synthesis by peritoneal mesothelial cells. Kidney Int.

[32] Doerner AM, Zuraw BL (2009). TGF-beta1 induced epithelial to mesenchymal transition (EMT) in human bronchial epithelial cells is enhanced by IL-1beta but not abrogated by corticosteroids. Respir Res.

[33] Forino M, Torregrossa R, Ceol M, Murer L, Della Vella M, Del Prete D (2006). TGFbeta1 induces epithelial-mesenchymal transition, but not myofibroblast transdifferentiation of human kidney tubular epithelial cells in primary culture. Int J Exp Pathol.

[34] Wang Y, Le Y, Xue JY, Zheng ZJ, Xue YM (2016). Let-7d miRNA prevents TGF-beta1-induced EMT and renal fibrogenesis through regulation of HMGA2 expression. Biochem Biophys Res Commun.

[35] Wu J, Huang Q, Li P, Wang Y, Zheng C, Lei X (2019). MicroRNA-145 promotes the epithelial-mesenchymal transition in peritoneal dialysis-associated fibrosis by suppressing fibroblast growth factor 10. J Biol Chem.

[36] Humphreys BD, Lin SL, Kobayashi A, Hudson TE, Nowlin BT, Bonventre JV (2010). Fate tracing reveals the pericyte and not epithelial origin of myofibroblasts in kidney fibrosis. Am J Pathol.

[37] Kriz W, Kaissling B, Le Hir M (2011). Epithelial-mesenchymal transition (EMT) in kidney fibrosis: fact or fantasy?. J Clin Investig.

[38] Oba S, Kumano S, Suzuki E, Nishimatsu H, Takahashi M, Takamori H (2010). miR-200b precursor can ameliorate renal tubulointerstitial fibrosis. PLoS ONE.

[39] Li P, Chen Y, Juma CA, Yang C, Huang J, Zhang X (2019). Differential inhibition of target gene expression by human microRNAs. Cells.

[40] Liu L, Hu J, Yu T, You S, Zhang Y, Hu L (2019). miR-27b-3p/MARCH7 regulates invasion and metastasis of endometrial cancer cells through Snail-mediated pathway. Acta Biochim Biophys Sin.

[41] Luu K, Greenhill CJ, Majoros A, Decker T, Jenkins BJ, Mansell A (2014). STAT1 plays a role in TLR signal transduction and inflammatory responses. Immunol Cell Biol.

[42] Loh CY, Arya A, Naema AF, Wong WF, Sethi G, Looi CY (2019). Signal transducer and activator of transcription (STATs) proteins in cancer and inflammation: functions and therapeutic implication. Front Oncol.

[43] Zhang H, Chen F, Fan X, Lin C, Hao Y, Wei H (2017). Quantitative proteomic analysis on activated hepatic stellate cells reversion reveal STAT1 as a key regulator between liver fibrosis and recovery. Sci Rep.

[44] Ortiz-Munoz G, Lopez-Parra V, Lopez-Franco O, Fernandez-Vizarra P, Mallavia B, Flores C (2010). Suppressors of cytokine signaling abrogate diabetic nephropathy. J Am Soc Nephrol.

[45] Kaowinn S, Kaewpiboon C, Koh SS, Kramer OH, Chung YH (2018). STAT1HDAC4 signaling induces epithelial–mesenchymal transition and sphere formation of cancer cells overexpressing the oncogene, CUG2. Oncol Rep.

[46] Huang F, Wang Q, Guo F, Zhao Y, Ji L, An T (2019). FoxO1-mediated inhibition of STAT1 alleviates tubulointerstitial fibrosis and tubule apoptosis in diabetic kidney disease. EBioMedicine.

[47] Zhang Y, Zhao S, Wu D, Liu X, Shi M, Wang Y (2018). MicroRNA-22 promotes renal tubulointerstitial fibrosis by targeting PTEN and suppressing autophagy in diabetic nephropathy. J Diabetes Res.

[48] Zhu Y, Xu J, Liang W, Li J, Feng L, Zheng P (2019). miR-98-5p alleviated epithelial-to-mesenchymal transition and renal fibrosis via targeting Hmga2 in diabetic nephropathy. Int J Endocrinol.

[49] Chevalier RL (2006). Pathogenesis of renal injury in obstructive uropathy. Curr Opin Pediatr.

[50] Chevalier RL (2006). Specific molecular targeting of renal injury in obstructive nephropathy. Kidney Int.

[51] Zhou X, Bai C, Sun X, Gong X, Yang Y, Chen C (2017). Puerarin attenuates renal fibrosis by reducing oxidative stress induced-epithelial cell apoptosis via MAPK signal pathways in vivo and in vitro. Ren Fail.

[52] Xu X, Fu XY, Plate J, Chong AS (1998). IFN-gamma induces cell growth inhibition by Fas-mediated apoptosis: requirement of STAT1 protein for up-regulation of Fas and FasL expression. Can Res.

[53] Song J (2007). EMT or apoptosis: a decision for TGF-beta. Cell Res.

